# Glomerulonephritis associated with systemic sclerosis: a case report

**DOI:** 10.1186/s13256-022-03727-7

**Published:** 2023-02-08

**Authors:** Sepehr Nayebirad, Alireza Ramandi, Fatemeh Nili, Reza Atef-Yekta, Zahra Tamartash, Samira Salehi, Hoda Kavosi

**Affiliations:** 1 Students’ Scientific Research Center (SSRC), Tehran University of Medical Sciences, Tehran, Iran; 2 School of Medicine, Tehran University of Medical Sciences, Tehran, Iran; 3 Department of Pathology, Cancer Institute, Imam Khomeini Hospital Complex, Tehran University of Medical Sciences, Tehran, Iran; 4 Department of Anaesthesiology,, Tehran University of Medical Sciences, Tehran, Iran; 5 Rheumatology Research Center, Tehran University of Medical Science, P.O. Box 1411713137, Tehran, Iran

**Keywords:** Systemic sclerosis, Glomerulonephritis, ANCA, Vasculitis

## Abstract

**Background:**

Systemic sclerosis is a multiorgan autoimmune disease that can overlap with other rheumatologic disorders; however, co-occurrence with antineutrophil cytoplasmic antibody-associated vasculitis is rare.

**Case presentation:**

A 39-year-old Persian female patient with systemic sclerosis according to American College of Rheumatology/European League Against Rheumatism 2013 criteria with a disease duration of 6 years was admitted to the hospital due to a rise in creatinine level in July 2021. She had complaints of nasal speech and feeling of nasal perforation. The first symptoms of antineutrophil cytoplasmic antibody-associated vasculitis had started 5 years earlier with palpable purpura in the lower limbs, hemoptysis, and positive perinuclear (p)-antibody-associated vasculitis level (> 300 AU/mL). Still, the diagnosis was not achieved due to the patient's reluctance to undergo a biopsy. She was treated with azathioprine (150 mg/day) and prednisolone (10 mg/day) during the 5-year follow-up. Her renal biopsy results showed cortical renal tissue with a cellular crescent in more than 50% of the specimen, rupture of the Bowman capsule and the glomerular basement membrane, peri-glomerular inflammation, and mild tubular atrophy in microscopic examinations. The immunofluorescence study resulted in a granular pattern of immune deposits along the glomerular basement membrane, mesangial tissue, and tubular basement membranes.

**Conclusion:**

We reported a rare case of comorbid systemic sclerosis and antineutrophil cytoplasmic antibody-associated vasculitis with nasal perforation. Her renal biopsy showed immune deposits along the glomerular basement membrane, mesangial tissue, and tubular basement membranes. Overlapping with other collagen vascular diseases can occur in rheumatology patients with uncommon manifestations. In systemic sclerosis, renal involvement in the form of glomerulonephritis is infrequent, and comorbid systemic lupus erythematosus or antineutrophil cytoplasmic antibody-associated vasculitis should be considered.

## Background

Systemic sclerosis (SSc) is a multiorgan autoimmune disease with skin and organ fibrosis [1]. It is a rare condition with a reported prevalence of 7.2–33.9 and 13.5–44.3 per 100,000 individuals in Europe and North America, respectively [2]. Overlap SSc, defined as the co-occurrence of SSc with other immune diseases such as rheumatoid arthritis (RA), Sjogren's syndrome, and systemic lupus erythematosus (SLE), is not an infrequent finding [3]. Elevated collagen synthesis and vascular fibrosis in the lungs, heart, gastrointestinal system, and kidneys characterize the multiorgan involvement in SSc patients [3]. The primary type of renal involvement in SSc is scleroderma renal crisis (SRC) with histopathological features of a thrombotic microangiopathic process and intimal accumulation of myxoid material [4]. Kidney involvement other than SRC is rare in SSc patients [5]; however, other types of renal involvement have also been reported in SSc patients with comorbid autoimmune diseases [6–8].

One notable but uncommon autoimmune disease co-occurring with SSc is antineutrophil cytoplasmic antibody (ANCA)-associated vasculitis (AAV) [6]. Kidney involvement is one of AAV's major manifestations, with 77 to 85% of the patients estimated to develop glomerulonephritis in the disease course [9, 10]. Renal biopsy of AAV patients with glomerulonephritis usually shows few or no immune deposits in the glomeruli (pauci-immune glomerulonephritis) [6–8]. The deposition of immune complexes such as immunoglobulin (Ig) and complement in the mesangium and glomerular basement membrane (GBM) is rarely observed [11, 12]. Nevertheless, the renal biopsy results in AAV are in stark contrast to that of the SRC.

As a result, the present study reports a unique case of a 39-year-old female patient with comorbid SSc and AAV. The patient presented with elevated creatinine levels and nasal speech. In addition, her renal biopsy demonstrated cellular crescents and a granular pattern of immune deposits along the GBM and mesangial tissue, which is an unusual finding in SRC or AAV.

## Case presentation

A 39-year-old Persian female patient with SSc according to American College of Rheumatology (ACR)/European League Against Rheumatism (EULAR) 2013 criteria with a disease duration of 6 years was admitted to the hospital in July 2021 due to a rise in creatinine levels.

*Initial SSc diagnosis:* SSc diagnosis was established in 2015. According to the patient's past medical history, the primary manifestations at diagnosis were a diffuse pattern of skin stiffness (Rodnan skin score [RSS] = 24), Raynaud's phenomenon, abnormal nail-fold capillaries, telangiectasia, anti-Scl 70 level of 70 (normal < 300), and antinuclear antibody (ANA) level of $\frac{1}{640}$ (normal < $\frac{1}{80}$). Erythrocyte sedimentation rate (ESR), C-reactive protein (CRP) levels, and creatinine levels were normal. The test results were negative for the lupus serology panel (anti-double-stranded DNA [anti-dsDNA], anti–Sjögren's-syndrome-related antigen A [anti-SSA/Ro], anti–Sjögren's-syndrome-related antigen B [anti-SSB/La], anti-nuclear ribonucleoprotein [anti-nRNP], and anti-phospholipid syndrome antibodies). The high-resolution computed tomography (HRCT) scan at diagnosis showed bilateral subpleural ground-glass involvement in 30% of both lungs and mild tractional bronchiectasis, indicating alveolitis. The patient was put on mycophenolate mofetil (2 g/day) and prednisolone (10 mg/day). She also received nifedipine 30 mg/day, fluoxetine 20 mg/day for Raynaud's, and pantoprazole 20 mg/day for gastroesophageal reflux. None of these medications routinely provoke perinuclear (p)-ANCA. Her forced vital capacity (FVC) and forced expiratory volume in 1 second (FEV_1_) remained stable throughout the treatment.

*Disease course:* After 9 months of treatment, the patient showed palpable purpura in the lower limbs and hemoptysis. Laboratory test results, including creatinine levels (0.74 mg/dL), were normal other than high levels of p-ANCA (> 300 AU/mL) and the presence of microscopic hematuria (20–25 red blood cells [RBCs]/high-power field [HPF]). There were no signs of dysmorphic RBCs or proteinuria in the urinalysis (U/A), complement levels were normal, and the lupus serology panel was negative. The second HRCT showed no changes compared with the first, with no evidence of cavitary lung lesions. The patient was suspected of having AAV concurrent with systemic sclerosis, but she refused to undergo a renal biopsy. She was treated with 30 mg per day of prednisolone (0.5 mg/kg/day) along with rituximab induction therapy (1 g at 0 and 2 weeks, then every 6 months) for 1 year, followed by maintenance therapy with azathioprine (150 mg/day) and prednisolone (10 mg/day). Mycophenolate mofetil was not initiated due to the patient's desire for pregnancy. After 3 months of maintenance therapy, the patient showed decreased FVC and diffusing capacity for carbon monoxide (DLCO) during the follow-up. The HRCT showed evidence of active ground-glass opacity and pulmonary fibrosis. The p-ANCA titer decreased after rituximab induction therapy, reaching 32; however, it did not return to normal levels (normal < 5). Azathioprine was stopped and substituted with mycophenolate mofetil (2 gm/day), along with tapering of prednisolone dosage to 7.5 mg/day. The patient's pulmonary function tests remained stable for the next 3 years.

In her follow-up visit in September 2020, laboratory tests showed ESR of 13 mm/hour, CRP less than 6 mg/L, normal U/A, and normal creatinine levels (0.8 mg/dL). Because of the COVID-19 pandemic, the patient did not attempt any follow-ups until July 2021, during which she complained of nasal speech and a feeling of nasal perforation. The patient declared that she had complied with the maintenance treatment during the pandemic. The nasal septum perforation was proven in the otolaryngology exam. The laboratory test results showed ESR 80 mm/hour, CRP 8 mg/L, serum creatinine 2 mg/dL, p-ANCA level (> 300 AU/mL), anti-Ro antibody 29 (normal < 18), and ANA $\frac{1}{160}$. The U/A showed 25–30 RBCs/HPF and 2–4 white blood cells (WBCs)/HPF, dysmorphic RBCs, and a 24-hour urine protein level of 3500 mg. She was not in her menstrual period. The anti-dsDNA antibody was negative, and the C3/C4 complement levels were normal. Her HRCT demonstrated tractional bronchiectasis, honeycombing, and 50% lung involvement, without any signs of active alveolitis. Because of glomerulonephritis, the patient underwent a renal tissue biopsy. She was treated with three 1-g pulses of methylprednisolone for three consecutive days, followed by a 1-g pulse of cyclophosphamide. She was discharged after 3 days with oral prednisolone (1 mg/kg/day) and a decrease in creatinine levels (1.7 mg/dL from 2 mg/dL).

Because the treatment occurred in the midst of the COVID-19 pandemic, a COVID-19 polymerase chain reaction (PCR) was obtained from the patient during hospitalization which proved to be negative. The patient had no signs or symptoms indicating COVID-19 during her admission; however, 5 days after discharge, she reported fever, malaise, and cough. She was immediately readmitted to the hospital with suspicion of COVID-19 infection. The PCR test for COVID-19 was positive, and the patient had symptoms of dyspnea, tachypnea, and low oxygen saturation (SpO_2_) levels, which progressed to acute respiratory distress syndrome (ARDS). The patient was later intubated and expired in the hospital due to the COVID-19 infection.

**Fig. 1:**
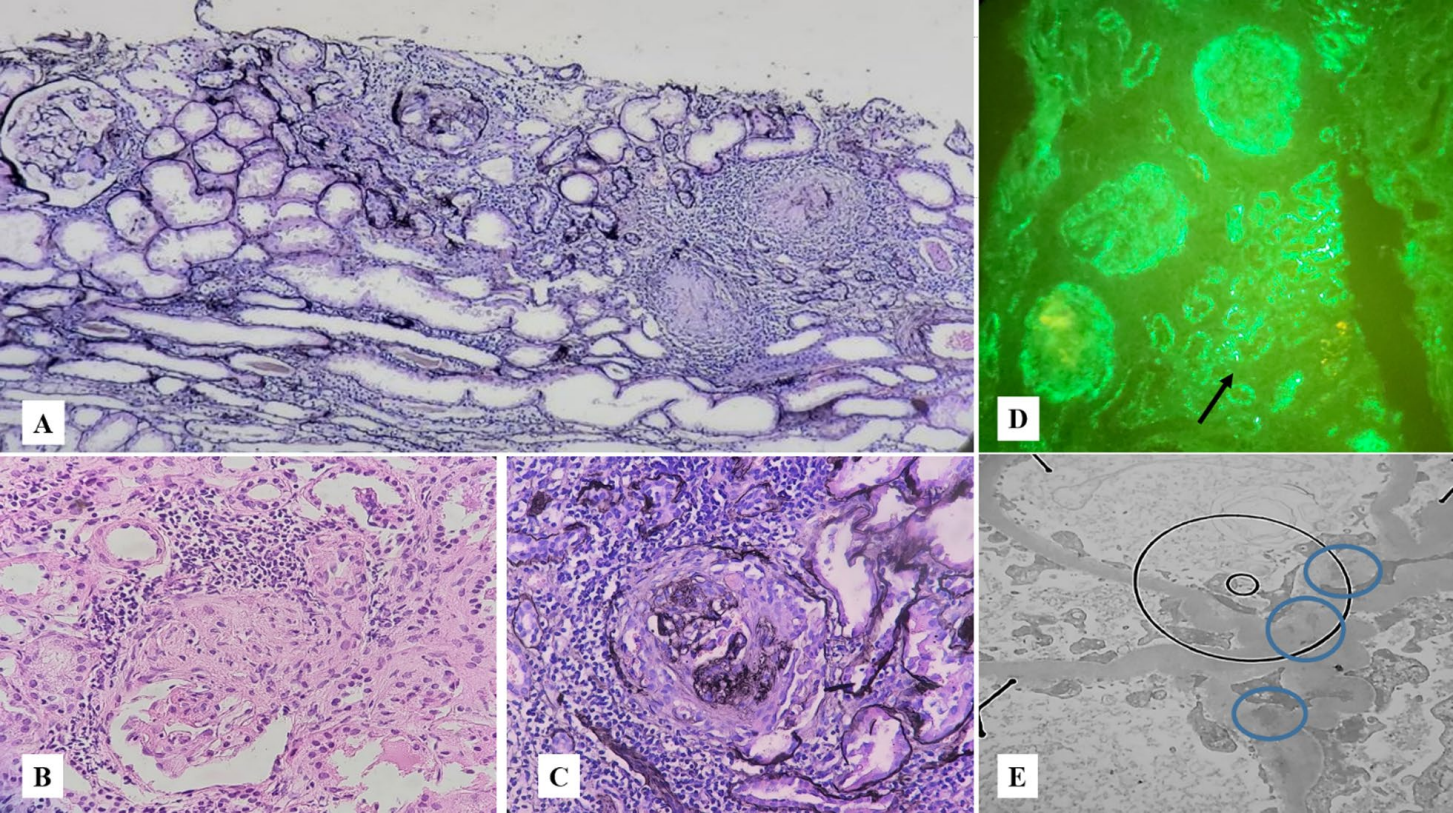
Microscopic examination shows renal cortical kidney tissue with cellular crescent, rupture of Bowman capsule and glomerular basement membrane, peri-glomerular inflammation, and mild tubular atrophy. **A** Special Jones’ stain (×100), **B** hematoxylin and eosin (H&E) stain (×400), **C** Jones’ stain (×400). **D** In the immunofluorescence study, a granular pattern of immune deposits was seen along the glomerular basement membrane and mesangium, as well as tubular basement membranes (black arrow) (immunoglobulin G [IgG], immunoglobulin A [IgA], complement component 3 [C3]: D: ×400). **E** Electron microscopic evaluation identified several mesangial electron dense deposits (blue circles). No subepithelial or subendothelial deposits were seen (×14,000)

The renal biopsy results were ready after the patient passed away, showing cortical renal tissue with a cellular crescent in more than 50% of the specimen, rupture of the Bowman capsule and the GBM, peri-glomerular inflammation, and mild tubular atrophy in microscopic examinations (Fig. 1). The biopsy specimen consisted of 15 glomeruli. Cellular and fibrocellular crescent were present in eight glomeruli, segmental sclerosis in four, and global sclerosis in two. The remaining glomerulus showed mild mesangial hypercellularity. The vessels were only atherosclerotic, and no evidence of vasculitis or thrombotic microangiopathy was observed. The immunofluorescence study resulted in a granular pattern of immune deposits along the GBM, mesangial tissue, and tubular basement membranes. The observed granular complexes in mesangial tissue were composed of IgG, IgA antibodies, and C3 protein. The intensity of IgG was 1 + , and trace amounts of others were found. Further pathologic evaluations using electron microscopy revealed several mesangial electron-dense deposits without signs of subepithelial or subendothelial depositions (Fig. 1).

## Discussion

SSc is characterized by vascular and fibrotic lesions affecting multiple organs, including the lungs, heart, and kidneys. Many rheumatologic diseases, such as SLE (SLE), Sjögren's syndrome, rheumatoid arthritis, and polymyositis/dermatomyositis, have been reported to overlap with SSc [13]. Although rarely reported, AAV is another disease that can co-occur with SSc. In 2013, a review study indicated that only 51 cases of AAV overlapping with SSc were reported, mainly consisting of microscopic polyangiitis or renal-limited vasculitis [7]. This number has since barely increased.

Renal manifestations of SSc are most commonly proliferative vasculopathy leading to SRC [4]. The observation of glomerulonephritis in SSc and signs of concurrent vasculitis of other origin is a rare but notable finding. Here, a 39-year-old woman was reported with an SSc diagnosis and concurrent clinical and laboratory signs of AAV (namely, hemoptysis, perforated nasal septum, palpable purpura in the lower limbs, and positive p-ANCA antibody). Her renal biopsy showed granular depositions of IgG/IgA antibodies and C3 complement in mesangial tissue with crescentic glomerulonephritis and fibrinoid necrosis, which is not commonly expected in AAV patients [6–8]. The immunofluorescence and electron microscopy studies showed similar findings with immune complex deposits in GBM.

Our first possible diagnosis for the patient was SSc with overlapping AAV, even with the mentioned renal biopsy findings. Previous studies have indicated that although pauci-immune crescentic glomerulonephritis is the primary type of glomerular involvement in AAV, histopathology exam in some AAV patients demonstrates substantial Ig and complement deposition in the mesangium and GBM [11, 12]. In addition, immune deposits have been associated with a more severe form of AAV and more severe proteinuria [11, 12]. One study reported average urine protein of 5.4 g/24 hours in eight AAV patients with immune deposits in renal biopsy. In contrast, proteinuria in pauci-immune glomerular involvements is expected to be much less, around 1 g/24 hours [11]. The patient reported here had proteinuria of 3.5 g/24 hours, which is consistent with the above findings.

Comorbid SSc, AAV, and SLE were also suspected in the patient because of the presence of immune deposits in her renal biopsy. However, class III and IV SLE nephritis were ruled out due to the absence of subepithelial or subendothelial deposits and proliferative changes in the glomeruli. Class II SLE nephritis was another possibility, but crescents are rarely (if ever) observed in type II SLE nephritis, as they are usually a feature of class III or IV [14–16]. Crescentic glomerulonephritis, also known as rapidly progressive glomerulonephritis (RPGN), is identified as a rapid loss of renal function and laboratory findings of nephrotic syndrome that can lead to death in weeks or months if left untreated [17], which contrasts with the generally favorable prognosis observed in type II SLE nephritis [18]. Additionally, the patient did not have any other clinical (that is, characteristic rashes, oral ulcers, neurological disorders) or laboratory (that is, positive anti-dsDNA, positive anti-Sm) criteria of SLE [19]. A case series of 14 SSc patients with comorbid AAV reported that nearly half of their patients had features of overlap or mixed connective tissue diseases, with sicca symptoms and SLE features being the most common [20]. Still, they did not report any patients having renal biopsies suggestive of lupus nephritis. In our report, the patient had positive anti-Ro antibody results but showed no symptoms suggestive of sicca syndrome. We believe anti-Ro positivity was due to systemic sclerosis, as other studies have demonstrated that SSc patients with positive anti-Ro are at risk of earlier lung involvement [21].

ANCA positivity in SSc patients has been reported in 0–12% of the patients [7]. Nevertheless, a study of a cohort of 2200 SSc patients estimated the number of ANCA-positive SSc patients who developed AAV to be much lower, around 0.4% [6]. Moxey *et al*. proposed interstitial lung disease (ILD), pulmonary embolism, overlap syndrome, and synovitis as independent predictors of ANCA positivity in SSc patients [22]. In addition, ANCA positivity was associated with significantly increased mortality [22]. In our report, the HRCT of the patient showed 50% lung involvement and a possible overlap syndrome with SLE, but no synovitis was observed.

## Conclusion

In conclusion, we reported a rare case of comorbid SSc and AAV with nasal perforation. The patient’s renal biopsy showed immune deposits along the GBM, mesangial tissue, and tubular basement membranes. Overlapping with other collagen vascular diseases can occur in rheumatology patients with uncommon manifestations. In SSc, renal involvement in the form of glomerulonephritis is infrequent, and comorbid SLE or AAV should be considered. Finally, the presence of immune deposits in renal biopsy of SSc patients with various clinical and laboratory features of AAV should not deter rheumatologists from the AAV diagnosis.

## Data Availability

The data for the current case report is available upon request from the corresponding author.
